# Supplementary figures and images for: Adaptation of metabolite leakiness leads to symbiotic chemical exchange and to a resilient microbial ecosystem
Source: PLoS Comput Biol. 2021 Jun 23;17(6):e1009143. doi: 10.1371/journal.pcbi.1009143 (PMC8260005; doi:10.1371/journal.pcbi.1009143)

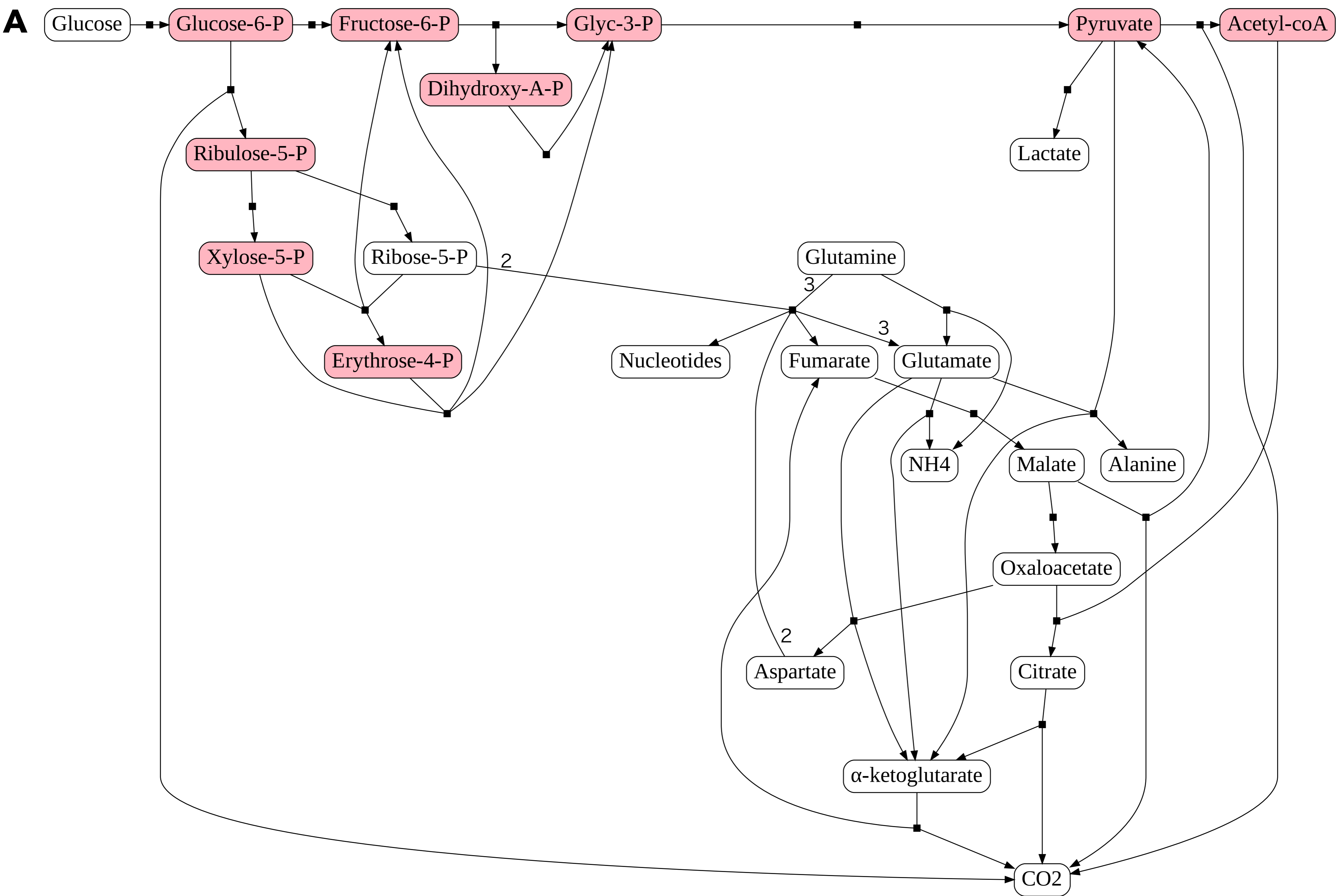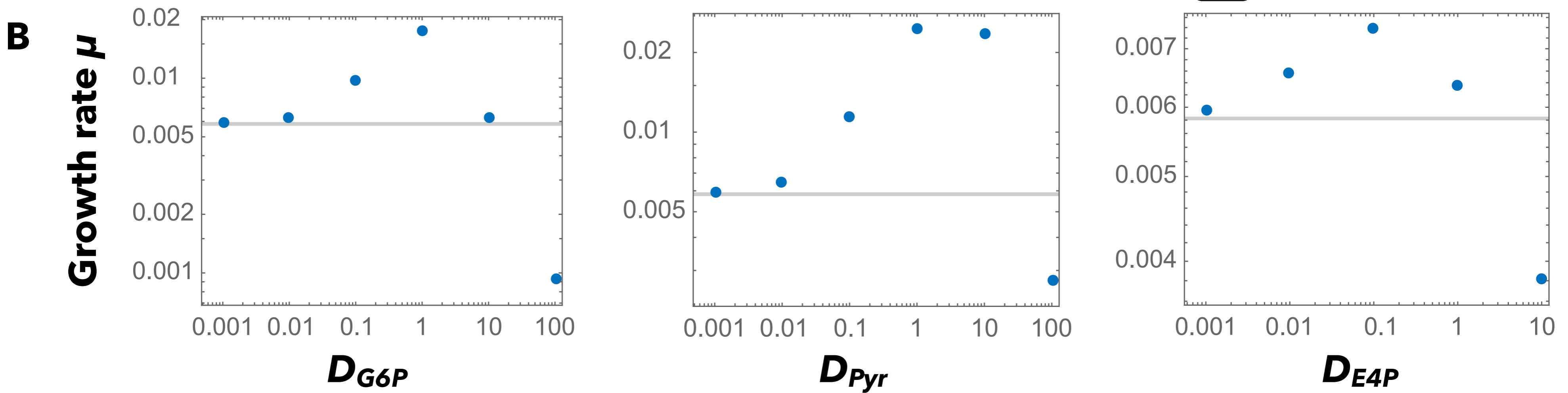

Supplement: S1 Fig — (A) The network structure of a minimal bioreaction model adopted from [73]. Each rounded box and filled square represents a metabolite and a reaction, respectively, and the numbers along some arrows indicate stoichiometric coefficients. For simplicity, all the rate constants are set at unity, and biomass is assumed to be made from nucleotides and alanine; accordingly, the growth rate is given by μ(x) ≡ xNucleotides xAlanine. Glucose and glutamine are externally supplied as nutrients: their diffusion coefficients DGlucose, DGlutamine and external concentrations xGlucose(env),xGlutamine(env) are fixed at 1.0, while the rest of the external concentrations x(env) are fixed at 0.0. The metabolites highlighted by pink are leak-advantage metabolites in the isolated condition, i.e., (moderate) leakage of them promote the cell growth, while the leakage of CO2, NH4, or lactate is neutral in our numerical simulations since they are at the bottom of the network. (B) Dependence of growth rate μ on the diffusion coefficient of some leak-advantage metabolites: Glucose-6-P (G6P), Pyruvate (Pyr), and Erythrose-4-P (E4P). The horizontal line in each panel exhibits the growth rate with no leakage of non-nutrient metabolites (μ = 0.0058). (PDF) [file pcbi.1009143.s001.pdf]

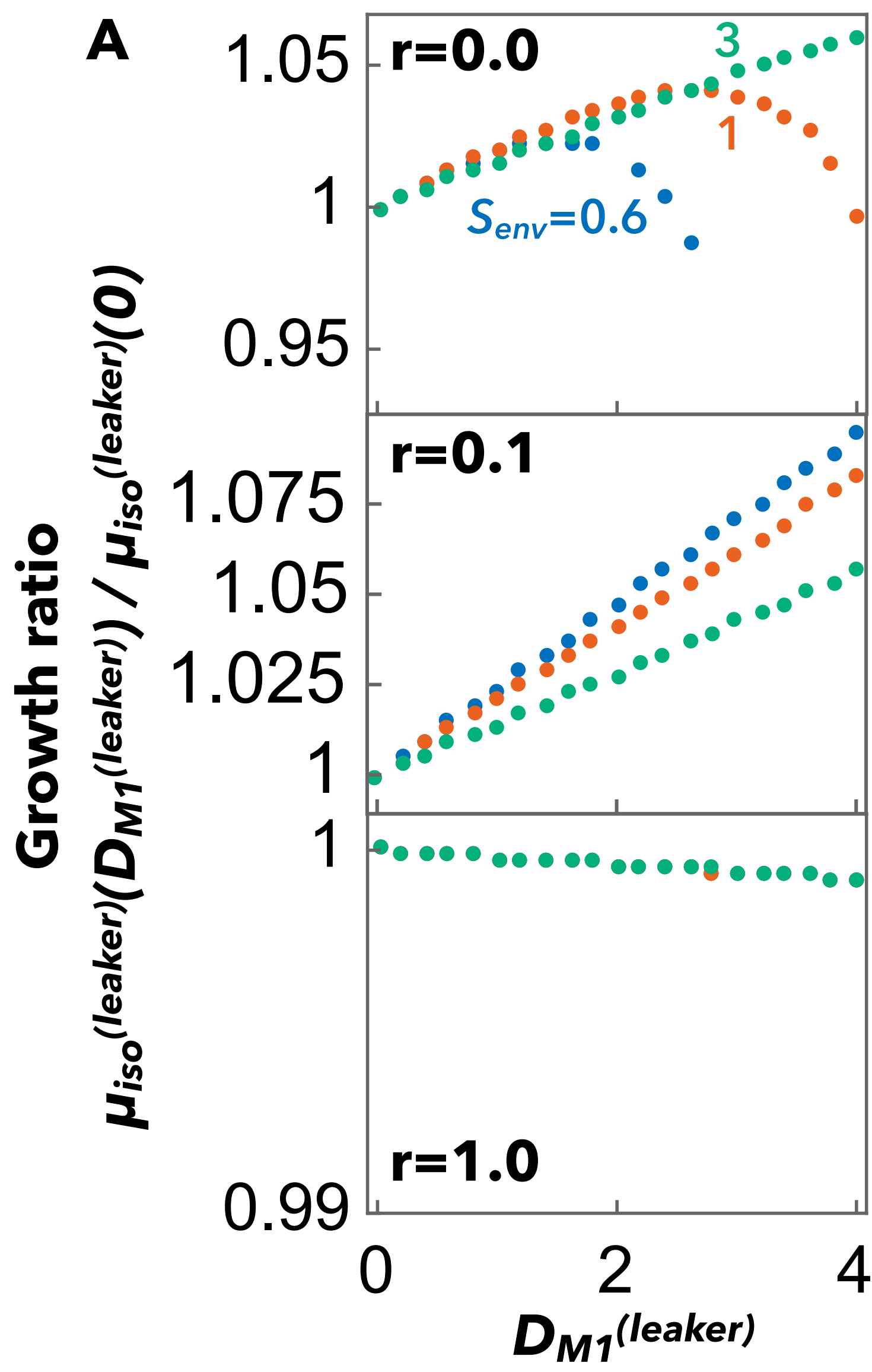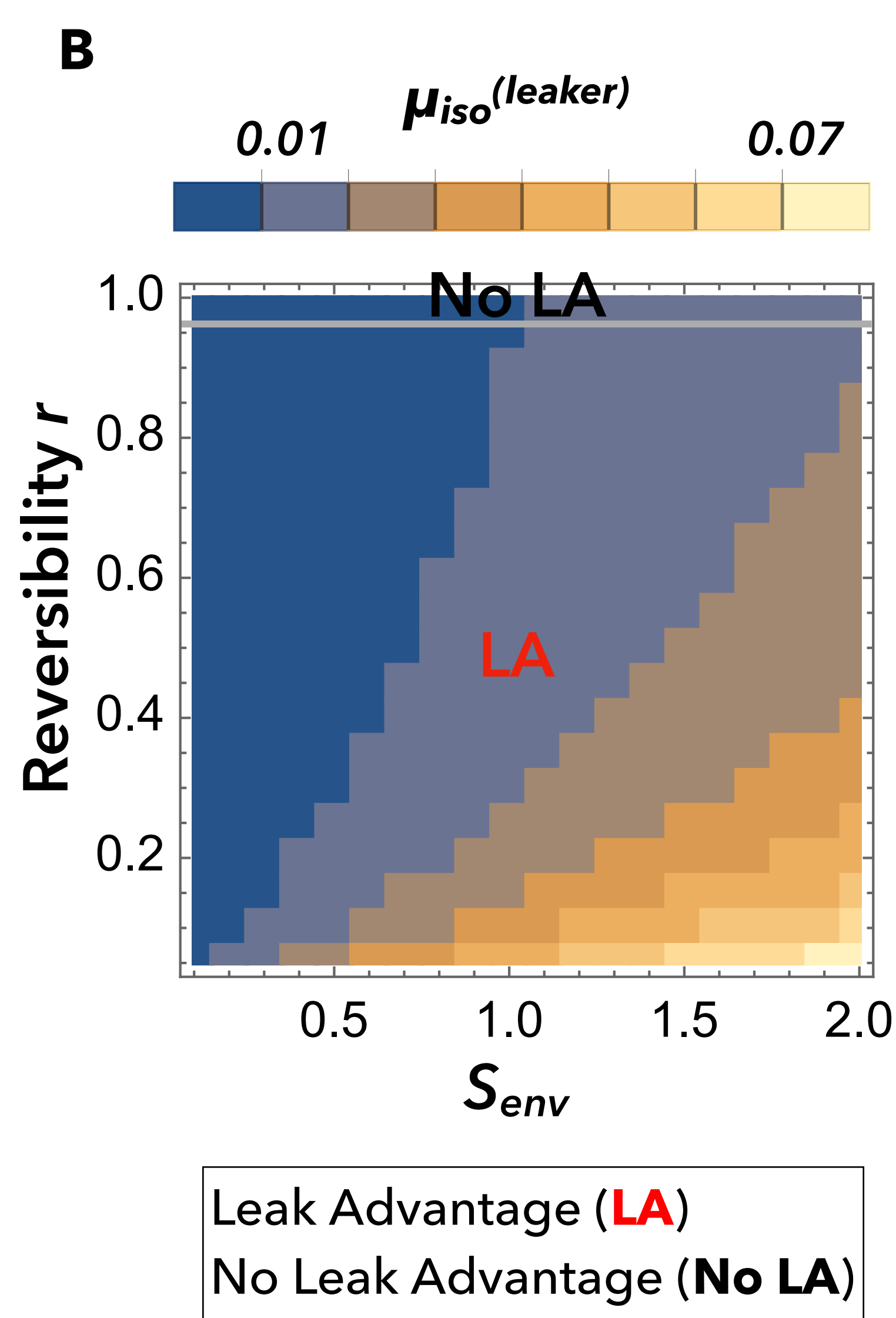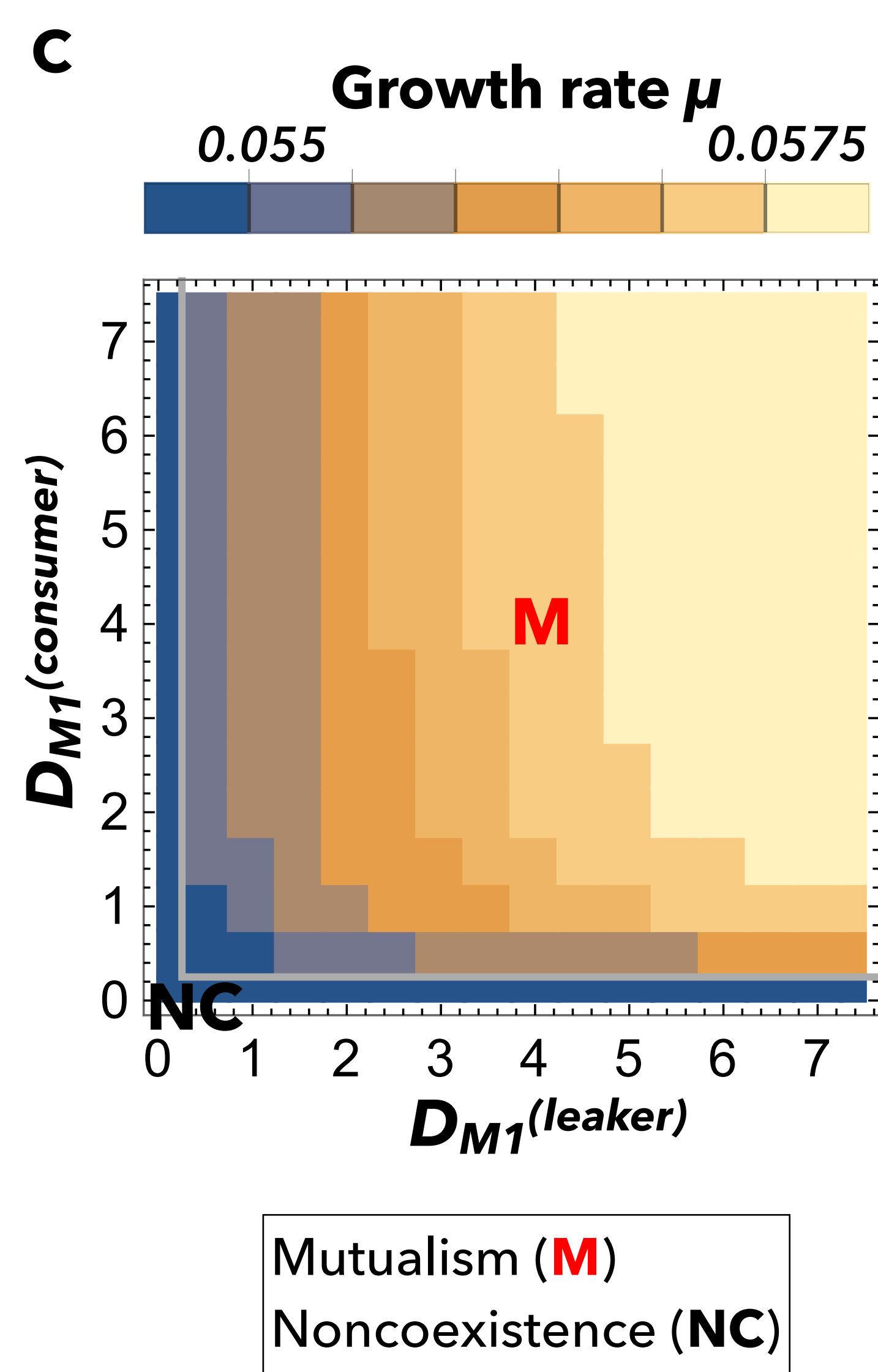

Supplement: S2 Fig — The network in Fig 2 is utilized, while the chemical reactions S + E → M1 + E and S → M2 are reversal with the strength r, i.e., kM1→S=rkS→M1 and kM2→S=rkS→M2. (A) Dependence of the isolated leaker’s growth rate on DM1 with the the reversibility r = 0.0, 0.1, 1.0. (B) Phase diagram of leak advantage of M1 for the isolated leaker cell species depending on the reversibility r and nutrient supply Senv. Regions LA (red) and NoLA (black) are delineated by gray lines and represent whether the leak advantage of M1 exists or not, respectively. (C) Phase diagram of symbiosis depending on DM1(leaker) and DM1(consumer) with the reversibility r = 0.01. Regions M (red) and NC (black) are delineated by gray lines and represent mutualism and noncoexistence, respectively. In the numerical simulations in (A)-(C), the rate constants are set as: kS→M1(leaker)=1,kS→M1(consumer)=0.3,kM1→rb(leaker)=kM1→E(leaker)=kS→M2(leaker)=1,kM1→rb(consumer)=kM1→E(consumer)=kS→M2(consumer)=2,kM2→BM(leaker)=kM2→BM(consumer)=0.01. The other parameters are set as Senv=1.0,DS(env)=10.0,DS=1.0,Venv=1.0,Rdeg=0.1. (PDF) [file pcbi.1009143.s002.pdf]

**A**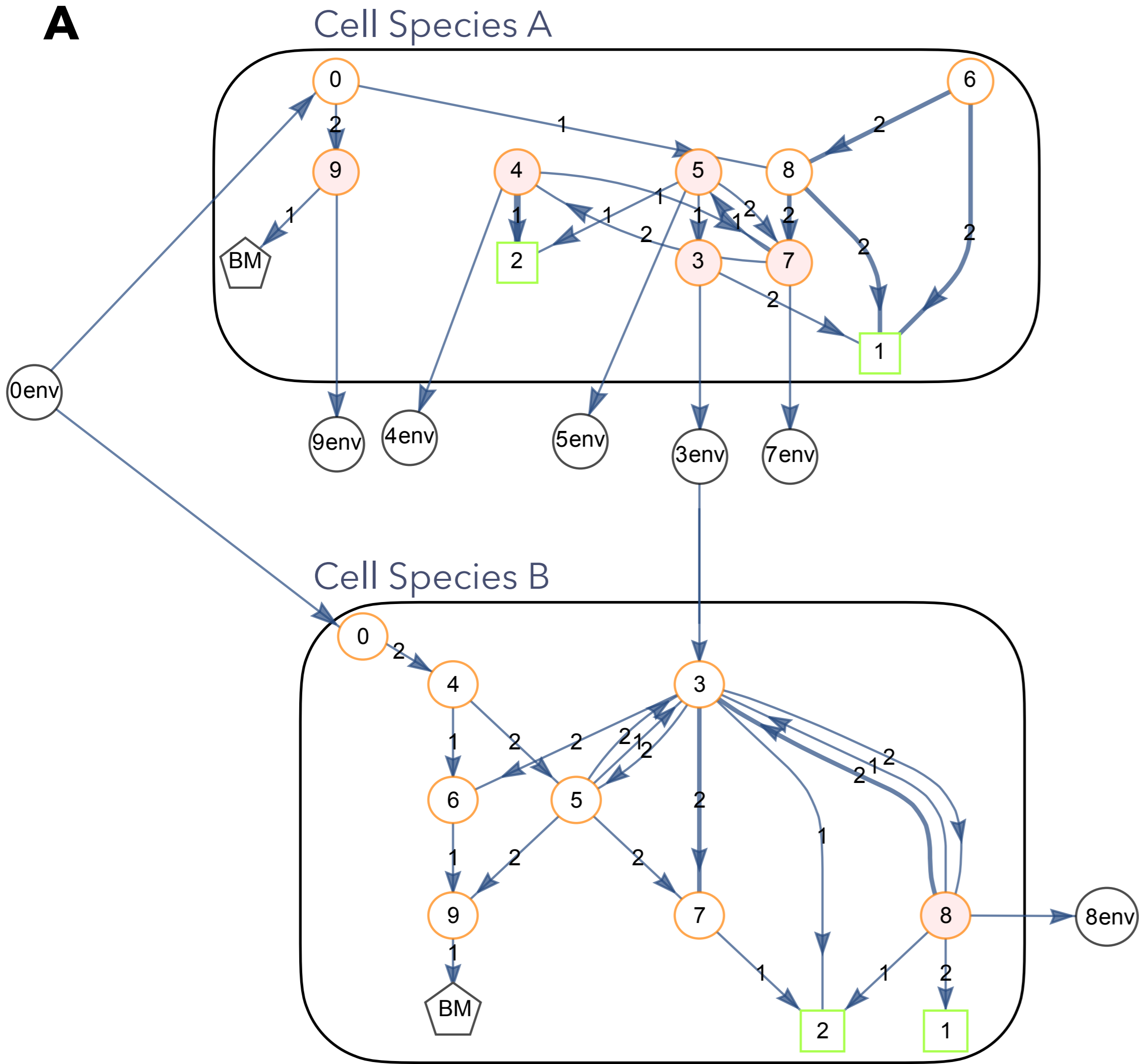**B**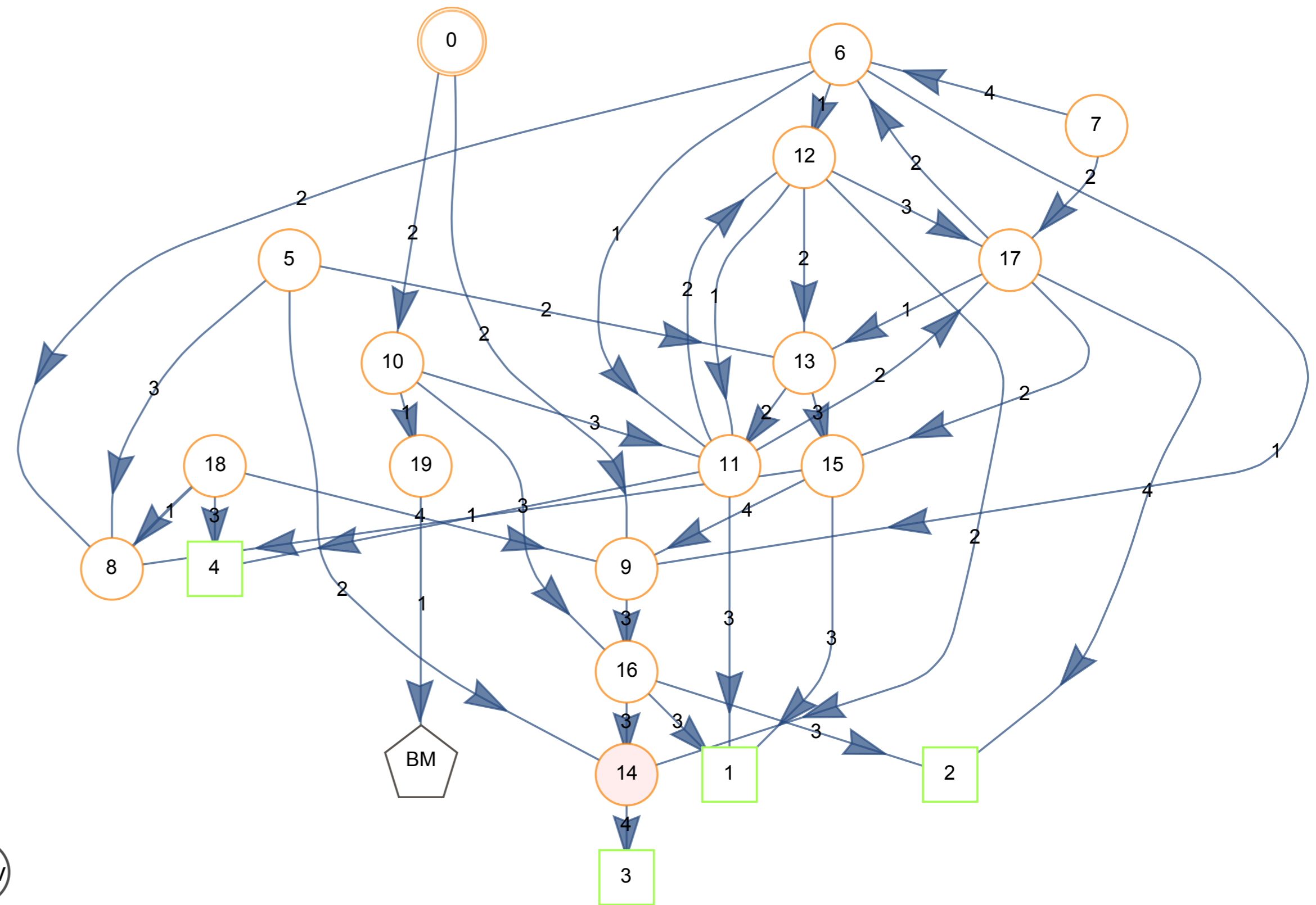

Supplement: S3 Fig — (A) Example of two-species symbiosis among randomly generated networks with n = 10, ρ = 2 and metabolite exchange between them. (B) Example of randomly generated networks with n = 20, ρ = 2 (cell species B in Fig 3C and 3D). The enzyme labeled on each arrow catalyzes the conversion of the metabolite at the arrowtail to the metabolite or enzyme at the arrowhead. Among n chemicals, chemicals 1 and nenzyme = n/5 are enzymes (green squares) and the nutrient chemical 0 and chemicals 3 to n − 1 are metabolites (orange circles). The leak-advantage metabolites for each cell species in isolation are highlighted by pink and adaptively leaked into the environment. (PDF) [file pcbi.1009143.s003.pdf]

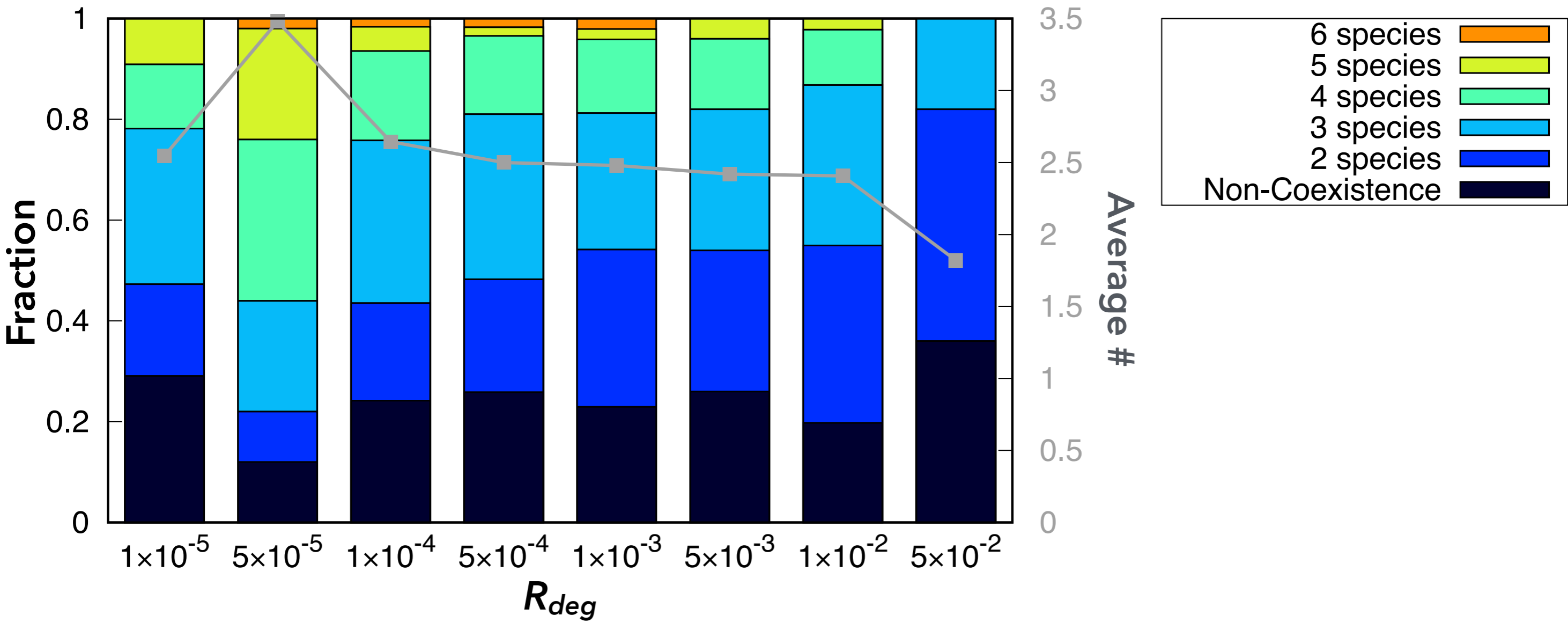

Supplement: S4 Fig — The colored bars show the frequency of symbiosis among two to six species (shown in different colors), whereas the black bars show noncoexistence. The frequency for each parameter set was calculated from 50 independent samples of N catalytic networks where the species with the fastest growth in isolation has a leak-advantage chemical in its reaction network. The other parameters are fixed: n=20,Senv=0.03,Venv=3.0,DS(env)=20.0,DS=1.0,nenzyme=n/5. (PDF) [file pcbi.1009143.s004.pdf]

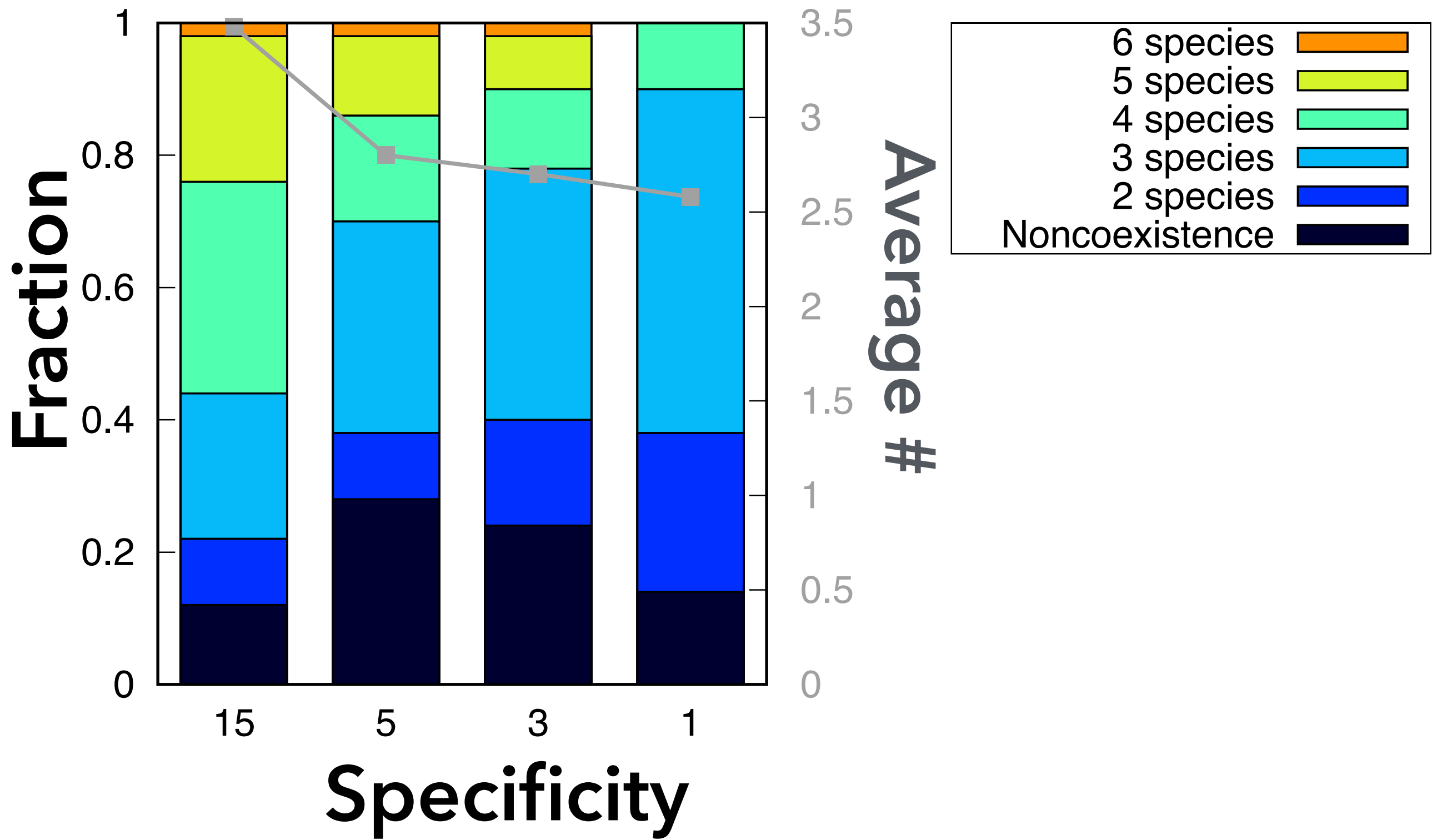

Supplement: S5 Fig — The value for “specificity” is defined as the degrees of freedom in the adaptation of the diffusion coefficients; for example, the case with specificity = 15 corresponds to the case in which each cell can alter the diffusion coefficients for all 15 non-nutrient metabolites independently; while in the case with specificity = 1, each cell species alters the diffusion coefficients of each metabolite all together across all metabolites, i.e., the diffusion coefficients of all metabolites are identical. When specificity equals an intermediate value 3 (5), each cell species has 3 (5) different values for the diffusion coefficients and alters the diffusion coefficients for 15/specificity = 5 (15/specificity = 3) non-nutrient metabolites together. The colored bars show the frequency of symbiosis with the number of coexisting species for two to six species (presented in different colors), whereas the black bars show noncoexistence. The frequency for each parameter set was calculated from 50 independent samples of N catalytic networks where the species with the fastest growth in isolation has a leak-advantage chemical in its reaction network. In the numerical simulation, the parameters were set to n=20,Senv=0.03,Venv=3.0,DS(env)=20.0,DS=1.0,Rdeg=5×10^-5^,nenzyme=n/5. (PDF) [file pcbi.1009143.s005.pdf]

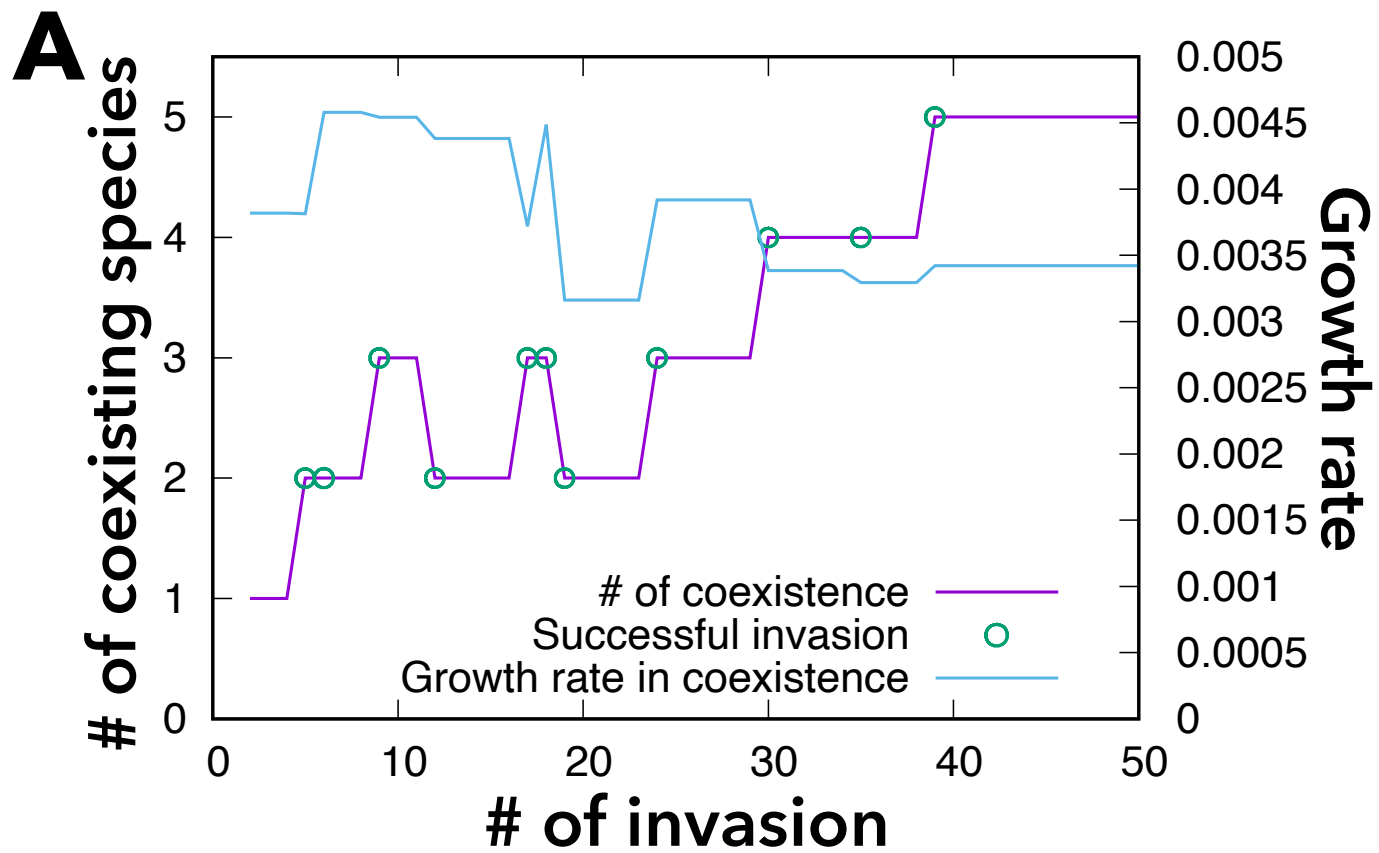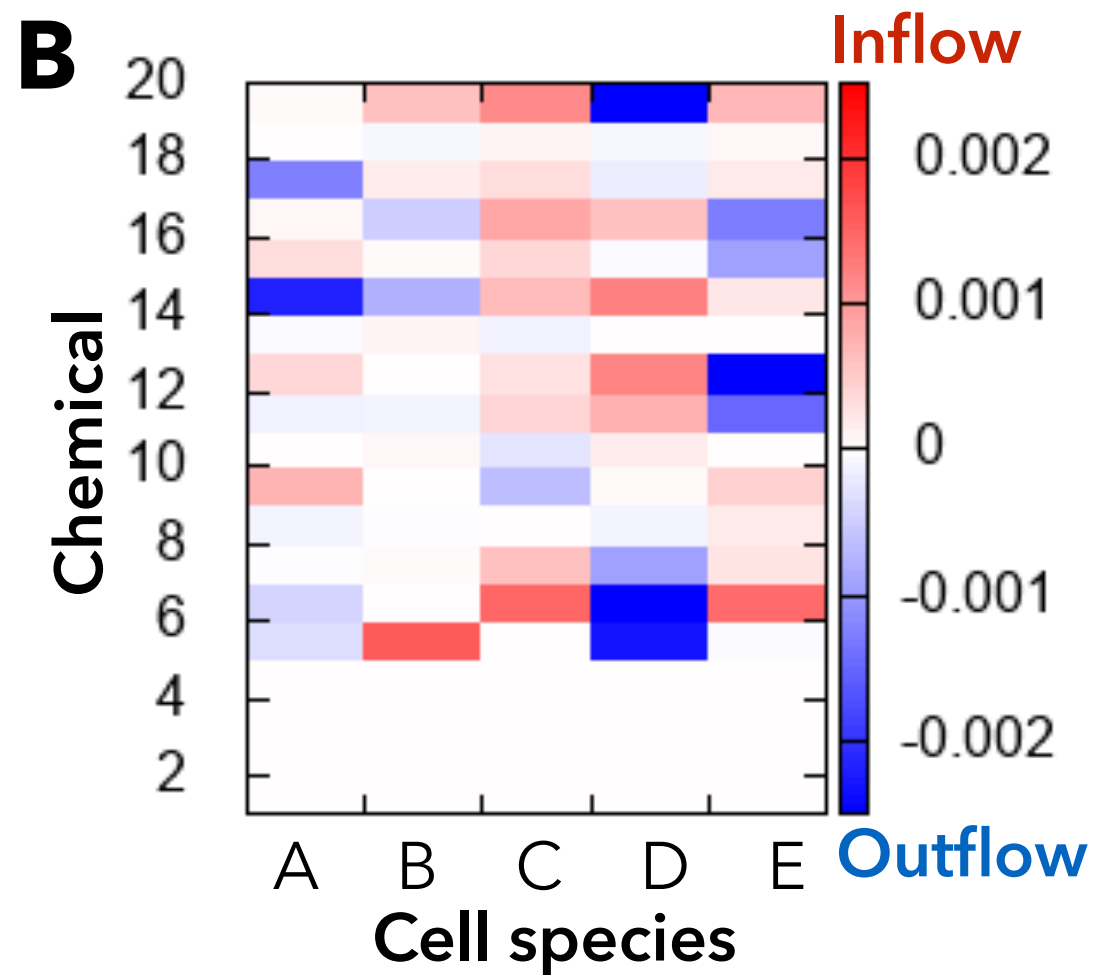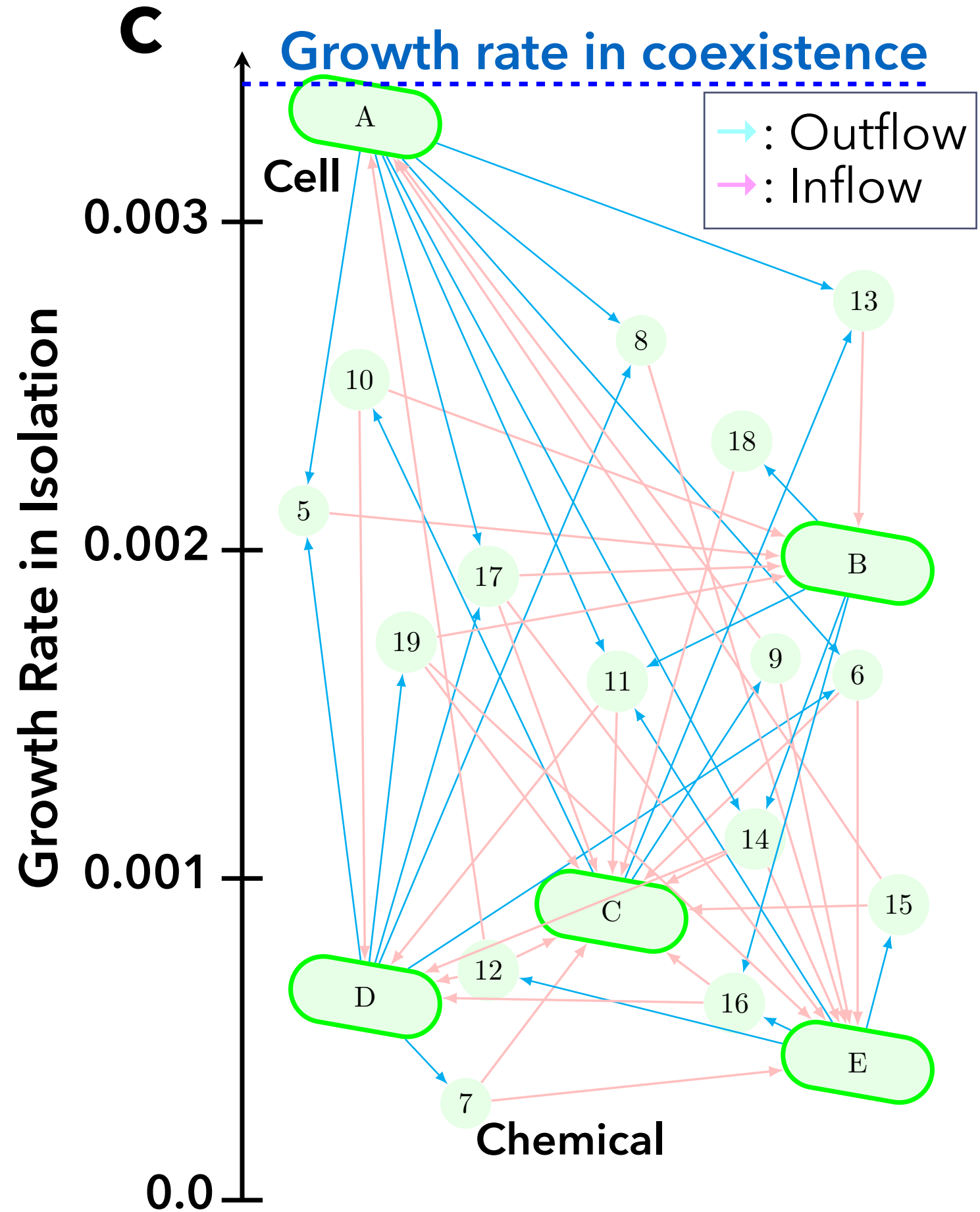

Supplement: S6 Fig — The diffusion coefficients of all the non-nutrient metabolites (chemicals nenzyme + 1 to n − 1) were randomly chosen from a uniform distribution [0.0: 1.0]. (A) Time series of the number of coexisting species by successful invasions of new species and the growth rate of cell species in coexistence. (B) Plot of leakage (blue) and uptake (red) fluxes of non-nutrient chemicals from each cell species A-E. (C) Structure of metabolic exchange among five species that originally have different growth rates in isolation. Cyan and pink arrows indicate the leakage and uptake of each chemical component, respectively. The growth rate while coexisting, μsymbiosis, is indicated on the top blue line. In the numerical simulation, the parameters were set to n=20,Senv=0.03,Venv=3.0,DS(env)=20.0,DS=1.0,Rdeg=5×10^-5^,nenzyme=n/5. (PDF) [file pcbi.1009143.s006.pdf]

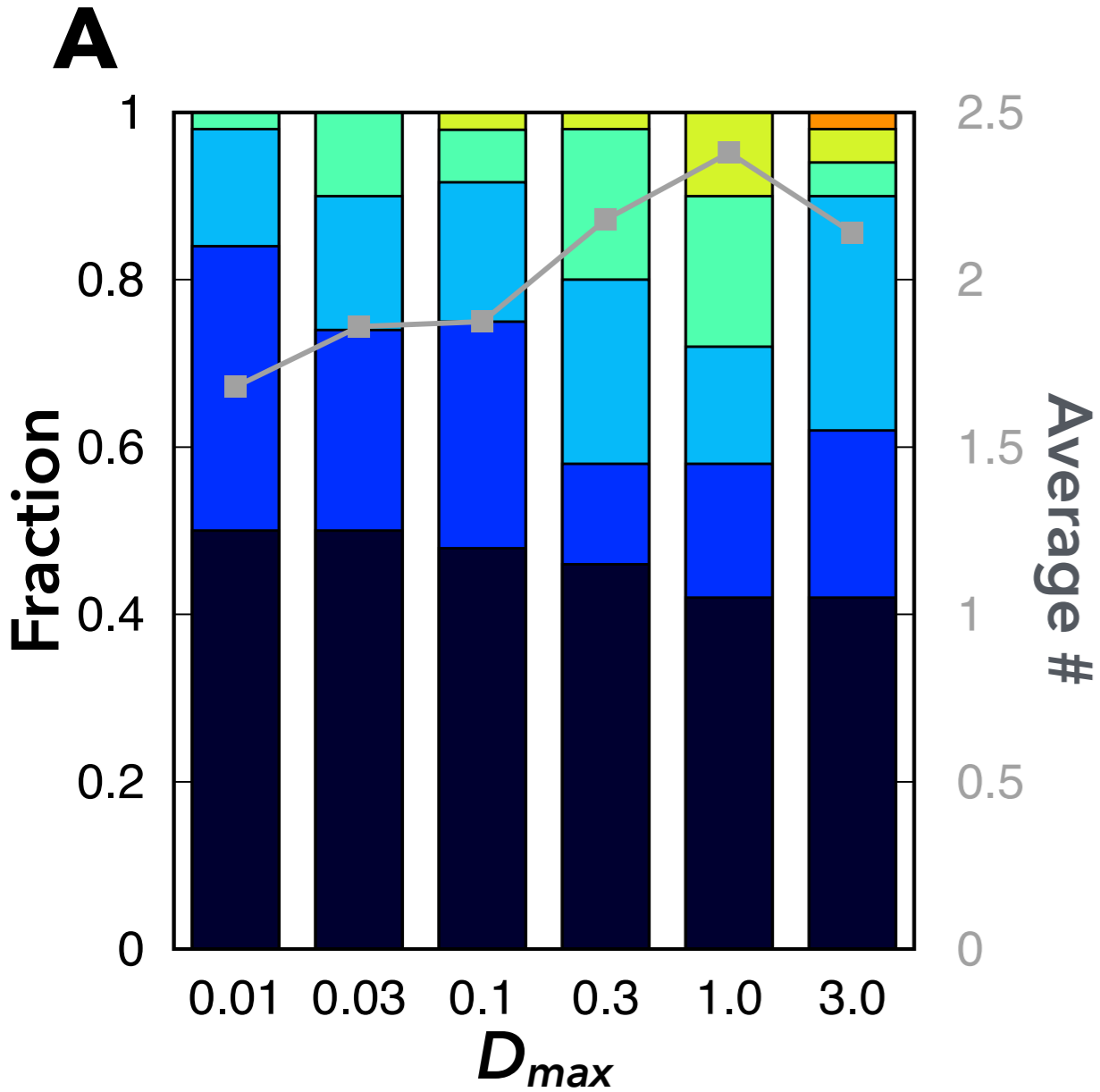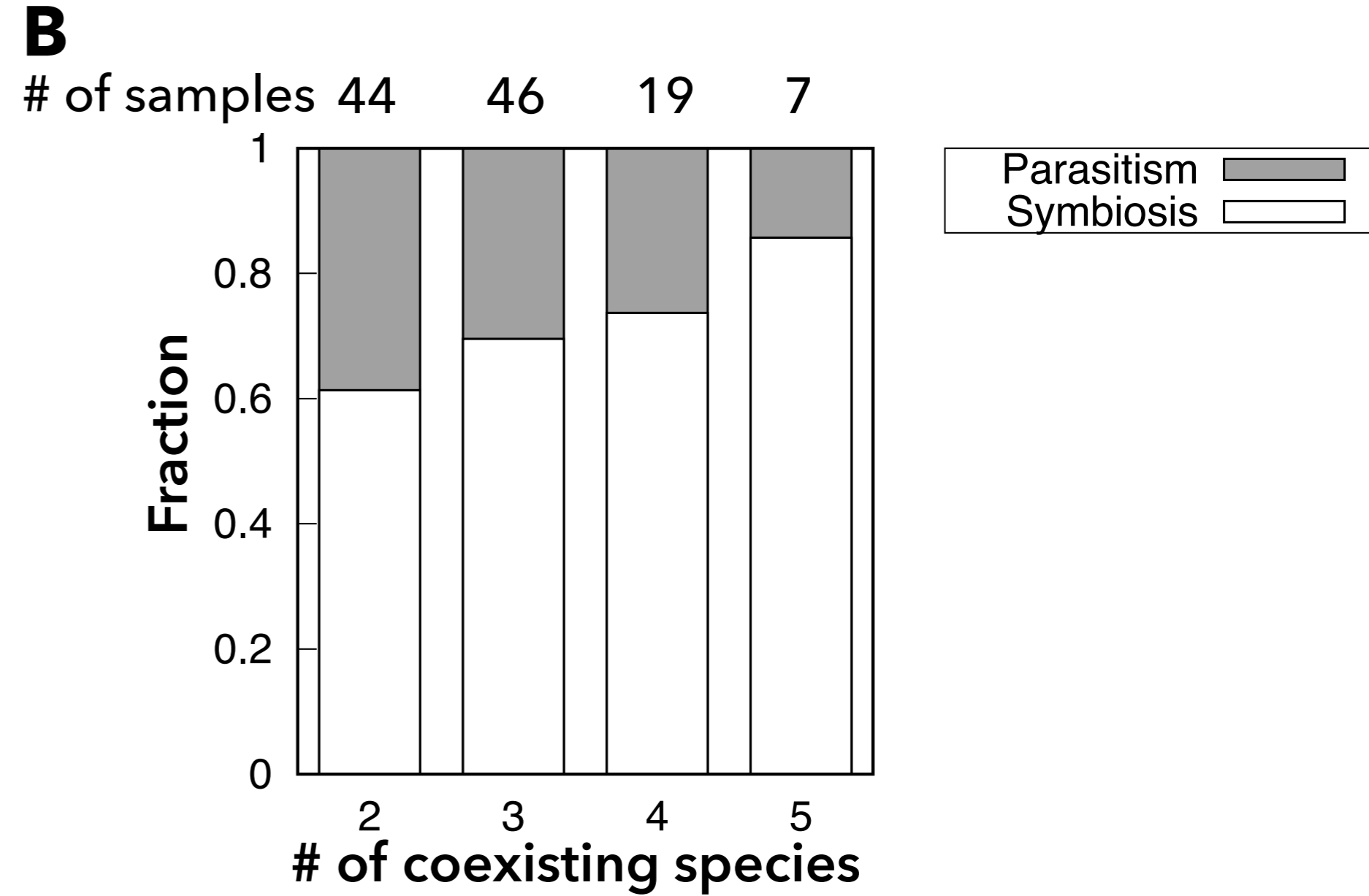

Supplement: S7 Fig — (A) Dependence of the frequency of coexisting species on the upper bound of randomly pre-fixed diffusion coefficients. The diffusion coefficients of non-nutrient metabolites (chemicals nenzyme + 1 to n − 1) were randomly chosen from a uniform distribution [0.0: Dmax]. The frequency for each parameter set was calculated from 50 independent samples of N randomly generated networks where the species with the fastest growth in isolation has a leak-advantage metabolite. (B) Relationship between the frequency of symbiosis/parasitism and the number of coexisting species (with various values of Dmax). In all the numerical simulations, the parameters are set at n=20,Senv=0.03,Venv=3.0,DS(env)=20.0,DS=1.0,Rdeg=5×10^-5^,nenzyme=n/5. (PDF) [file pcbi.1009143.s007.pdf]

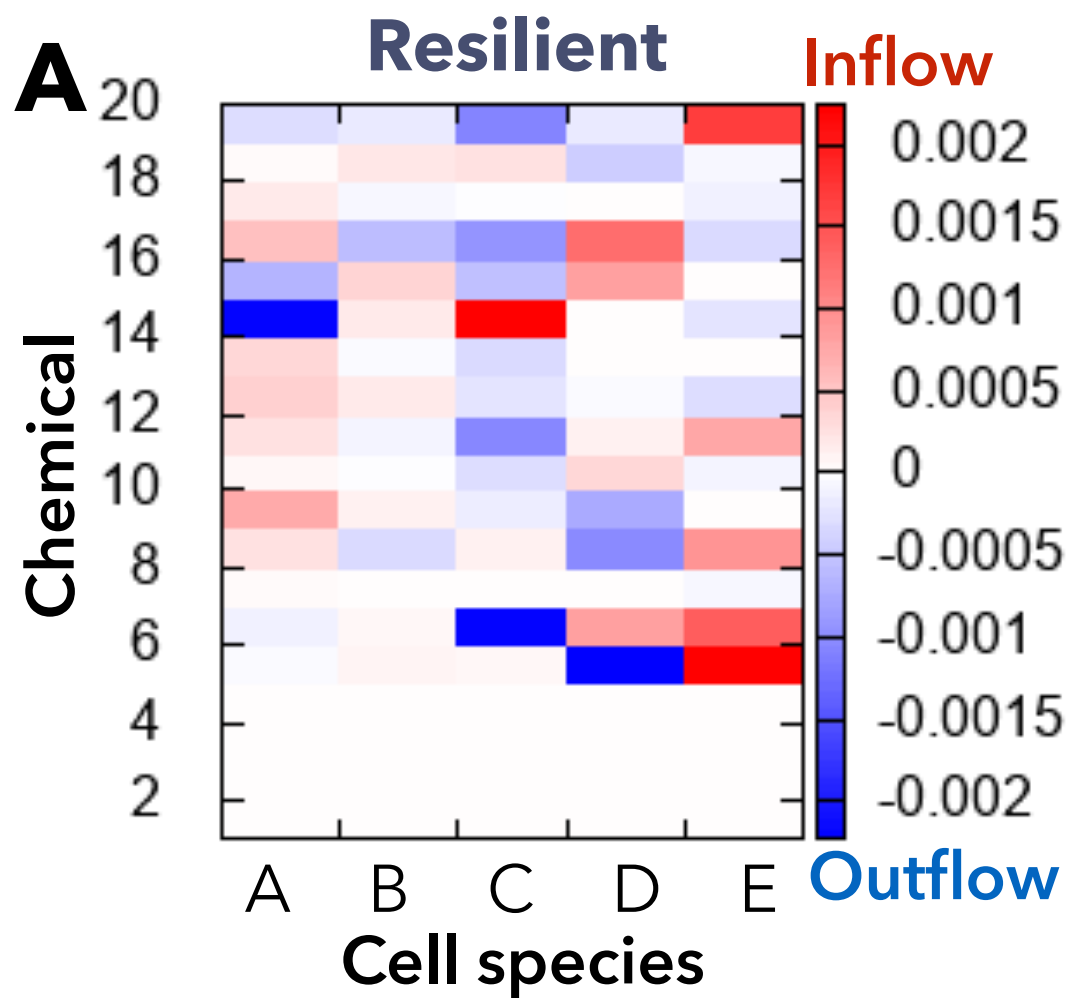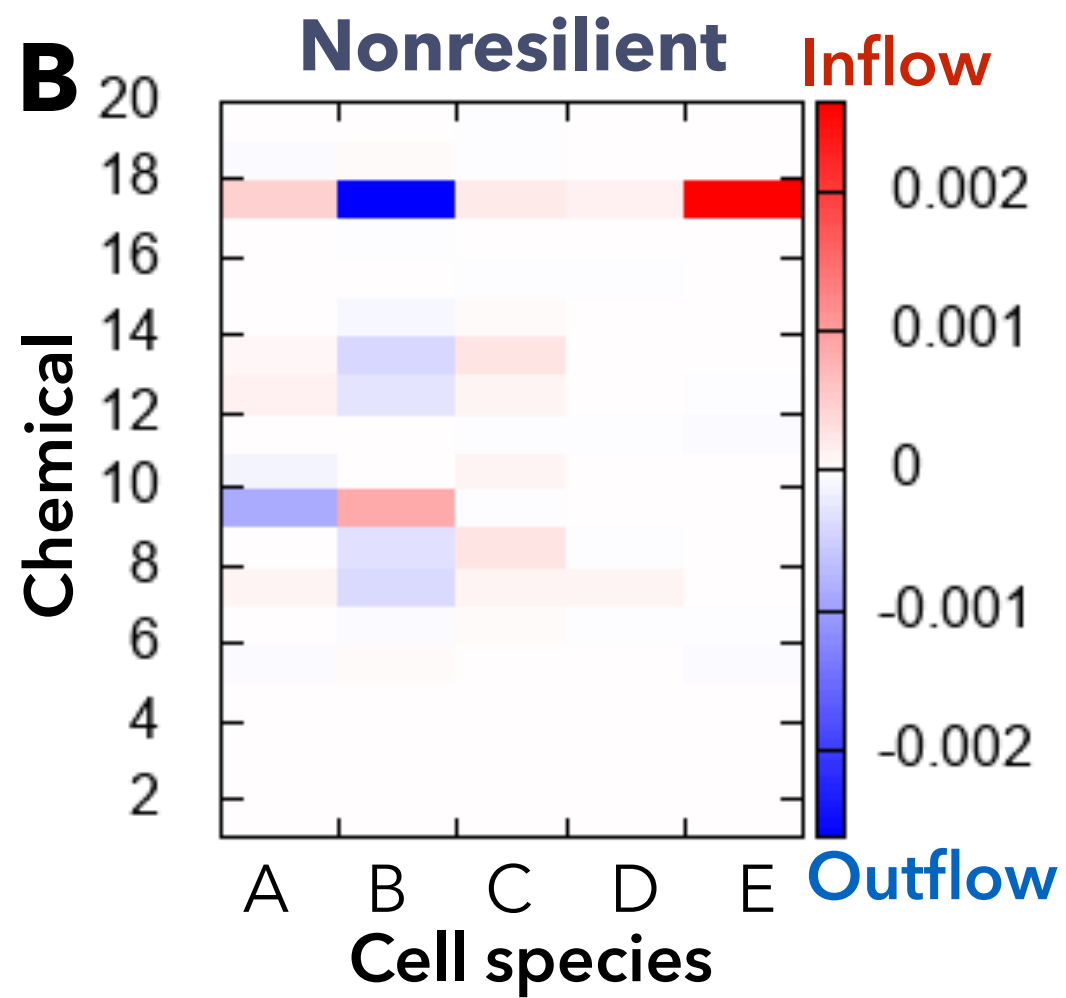

Supplement: S8 Fig — (A) The removal of any species does not cause the extinction of another species (average survival ratio is 1). (B) The removal of cell species B leads to the extinction of the other three cell species (C,D,E) leaving only cell species A in the community (average survival ratio is 0.8). Blue and red indicate the leakage and uptake of each chemical component, respectively. In both simulations, the parameters are set at n=20,Senv=0.03,Venv=3.0,DS(env)=20.0,DS=1.0,Rdeg=5×10^-5^,nenzyme=n/5. (PDF) [file pcbi.1009143.s008.pdf]
