# Supplementary material for: Adaptation of metabolite leakiness leads to symbiotic chemical exchange and to a resilient microbial ecosystem
Source: PLoS Comput Biol. 2021 Jun 23;17(6):e1009143. doi: 10.1371/journal.pcbi.1009143 (PMC8260005; doi:10.1371/journal.pcbi.1009143)
Supplement: Attachment — Submitted filename: Review.docx [file pcbi.1009143.s010.docx]

In this manuscript, Yamagishi et al. tackle an important problem of species coexistence in microbial ecosystems. They hypothesize that leakage of useful metabolites may be advantageous to the leaker as well as the receiver and using simulations show that leakage can indeed lead to community co-existence.

While their simulations are technically sound, I have several major issues with the correspondence of their model with real bacteria and therefore the validity of their conclusions. Prior to publication, the authors should address these concerns. Below, I point out my concerns.

1. The authors claim that if the leaked metabolite is not consumed, its concentration in the environment will increase and this will be detrimental to the leaker. However, no intuitive mechanism is presented for this detriment. I looked up their PRL 2020 to find out how this happens. The way I understand it is as follows: The leak advantage stems from the fact that the leaked metabolite (M1 in Fig. 2A) produces its own enzyme E which in turn reroutes the overall flux of the substrate S towards the leaked metabolite and away from biomass production. Therefore, it is advantageous for the organism to maintain low concentrations of M1 in the intracellular medium. Given that the intracellular and the extracellular medium are connected through a diffusion term, it is consequently advantageous to keep a low concentration of M1 in the extracellular medium as well. It appears to me that the symbiosis observed in their model relies on this mechanism therefore it is important to justify that this mechanism is indeed true. Can the authors point to papers where excess of internal metabolites (for example amino acids) leads to production of excess enzymes and therefore a lowered growth rate? If so, they should cite these papers. The authors at least should provide an intuitive explanation about why the leaker is at advantage (the way they did in their PRL paper)
2. The authors assume that some of the reactions in the model are reversible (diffusion across the membrane) while others are irreversible S -> M1. All biological reactions are essentially reversible. Importantly, the increase in S -> M1 flux that is presumably responsible for lower growth rate under conditions of high M1 may not be realized in a thermodynamically balanced situation where a high M1 may automatically decrease the S to M1 flux. As a broad comment, the authors should address the lack of thermodynamic equilibrium in their model and how introducing thermodynamic equilibrium may affect their phase diagrams.

Minor concerns:

1. The authors should provide a notation table. There are many quantities and it would be easy to follow the paper if the notations were clarified at the start
2. Fig. 3 caption mentions rho which is only defined in the appendix
3. Typo on line 129
