# Supplementary material for: Adaptation of metabolite leakiness leads to symbiotic chemical exchange and to a resilient microbial ecosystem
Source: PLoS Comput Biol. 2021 Jun 23;17(6):e1009143. doi: 10.1371/journal.pcbi.1009143 (PMC8260005; doi:10.1371/journal.pcbi.1009143)
Supplement: Attachment — Submitted filename: Responses to reviewer comments.docx [file pcbi.1009143.s011.docx]

**Reply to the reviewers**

**To Reviewer #1**

We would like to thank you for your positive evaluation and constructive comments and suggestions. Detailed below are the changes made to the manuscript based on your recommendations. Corrections and text that were added to the manuscript are indicated in red font in the main document and Supplementary Material.

1. Reviewer’s comment:

**“Reviewer #1: In their paper, Yamagishi, Saito, and Kaneko argue that metabolite secretion in mixed communities can lead to mutualism between “leaker” and “consumer” cells in a microbial community. This work contributes to a topic of considerable contemporary interest: What allows complex microbial communities to assemble, and what mathematical frameworks can be used to understand this process? The manuscript is quite well written and easy to follow. I have a small number of questions and comments:

-Line 107: “of the above equation”: Please include explicit equation numbers for clarity. ”**

Response:

We have explicitly stated the equation number (line 109 in the revised manuscript) as suggested.

2. Reviewer’s comment:

**“-Line 155: “randomly generated networks”: I understand that this is a model that is easy to implement computationally, however, can you provide some insight into whether this is an appropriate model for understanding shared metabolic networks in natural systems?**

**For example, have random networks like this been used to understand shared metabolites in the microbiome, etc.?”**

Response:

Regardless of the detailed structures of metabolic reaction networks, leaker-consumer mutualism can work as long as a species has the leak advantage chemical(s) and another can usefully consume the leaked (essential) metabolite(s). Thus, (as you indicated) randomness is not essential for leak advantage and leaker-consumer mutualism, and random networks were chosen purposely for the ease of numerical simulations.

We here want to emphasize that leak advantage generally appears without specific types of network structures. Besides, the results may be general in reaction networks with large degrees of freedoms as the frequency of leak advantage and metabolite-mediated coexistence increases with the number of chemicals *n*, and leak advantage is most likely to appear with a “realistic” degree of metabolic networks (ρ=1~2) (see our PRL 2020).

To further demonstrate the versatility of our theory, we carried out a numerical simulation with a more “realistic” metabolic network adopted from ref. 73, clearly distinct from random networks. Then, we confirmed the appearance of leak advantages of essential metabolites. We have added this result as Fig. S1.

Anyway, studying the behavior of more “realistic” metabolic networks will be an important part of future research. Interestingly, results of some previous experimental studies have suggested that actual microbes benefit from leaking certain essential metabolites (see e.g., ref. 9 in the manuscript).

We have added remarks on the above points to the Discussion and Results (lines 125-126 and 359-363).

3. Reviewer’s comment:

**“-Line 169: “After the addition of the new species”: It’s not clear to me that the model will yield the same result if the order of species introduction is altered. Can you comment on the importance of historical contingency (the order in which species are introduced) in your model?”**

Response:

The type of species that ultimately coexist (shown in Fig. 3 in the manuscript) can depend on the order in which new species are added, as is the case in actual microbial communities (see e.g., ref. 38 in the revised manuscript, which shows that the success or not of a species invasion often depends on the coexistence of other cohorts).

However, the statistics of the fraction of symbiosis due to leaker-consumer mutualism (shown in Fig. 4 in the manuscript) are independent of the order of the introduction, since the statistics are based on independent simulations with random metabolic networks; in this sense, our results are reproducible.

We had previously mentioned this point in the Discussion (lines 286-289 in the previous manuscript), but we have moved the statement to lines 194-196 in the Results now.

4. Reviewer’s comment:

**“-Line 184: “BQH”: This acronym doesn’t get used all that often. Writing out Black Queen Hypothesis would make the paper more readable. ”**

Response:

Following your suggestion, we have stopped using the acronym “BQH” throughout the paper.

5. Reviewer’s comment:

**“-Figure 3A: The random network is so large that’s is hard to interpret as a viewer. Perhaps a smaller network (like N = 6, as in panel D) would be more appropriate?”**

Response:

We agree with the reviewer. Based on your suggestion, we have replaced the example of a random network with n=20 with one that has n=10 in Fig. 3A. The example network with n=20 is now moved onto Fig. S2.

6. Reviewer’s comment:

**“-Line 231: “increases(S1)” Missing space.**

**-Line 242: “species that coexisted”: Missing an additional clause, such as: “species that coexisted with other species in the community” ”**

Response:

Thank you for the correction. We have fixed the mistakes at lines 240 and 251-252.

7. Reviewer’s comment:

**“-Line 296: “Microbial potlach”: I really like this phrase, but I wonder if more clarification is needed. My, very naïve understanding (I’m not an anthropologist!), is that potlaches were all about showing off your prestige, and not getting an immediate benefit from the act.**

**This seems to be different from your model, so a little more description would be useful before coining a term that may be confusing to many readers. ”**

Response:

Thank you for the comment. As you indicated, our theory does not completely correspond to potlatch in the human society. However, in this metaphor, we considered that the prestige or respect gained through gift-giving in potlatch could be considered as some kind of benefit.

Above all, in both the actual potlatch and “microbial potlatch,” people or microbes competitively increase their gift-giving rather than select the strategy to reduce gift-giving. Such similarity in trend seems interesting.

Following your suggestion, we have added a remark on the above points, especially on the difference between the actual potlatch and “microbial potlatch” (lines 303-305 in the revised manuscript).

8. Reviewer’s comment:

**“-Line 346: “is testable”: I agree that testing this model in a laboratory setting would be exciting. However, I wonder if in the interim you could provide some insight into whether the parameters used in your model are within a physiologically relevant range?**

**I know this can be challenging, but such data, however modest, would broaden the relevance of these findings, especially given that your model has a large number of parameters.”**

Response:

As rightly indicated, our model includes a large number of parameters, but please note that the leak advantage can appear with a wide range of parameters (over orders of magnitude) (for example, see Fig. 4BC). Here, it is reasonable to consider that the range of parameters with leak advantage includes a part of the physiological range.

In particular, the orders of the ratio of the medium volume to the total volume of microbes Venv=1~1000 and the degree of metabolic reaction networks ρ=1~2 seem to be relatively “realistic”: e.g., according to “Cell Biology by the Numbers” (ref. 74 in the main text) Chap. 2, p.85, Venv is about 1000 or smaller.

We have added remarks on these points to the Discussion (lines 360-361).

In the statement, by the term “testable,” we mean that operations to verify a leak advantage are experimentally practicable. Still, we need both theoretical and experimental studies in future to verify the symbiosis based on a leak advantage presented here.

9. Reviewer’s comment:

**“-SI material: The high-level description of the code is quite useful, however can you also provide details of how this was implemented, such as software and libraries used for solving these models? ”**

Response:

We implemented the 4th order Runge-Kutta method mainly by C++ to solve the ODEs in our manuscript, while we also utilized an ODE solver (NDSolve) in Mathematica to draw Fig. 2CD in the main text.

We have added a remark on this point into Text S1.

10. Reviewer’s comment:

**“-Code availability: Please provide a link to the code (e.g., a github page) used in your simulations.”**

Response:

We have provided a GitHub link to the C++ and Mathematica codes used in our simulations according to your suggestion.

**To Reviewer #2**

We would like to thank you for your positive evaluations and constructive comments and suggestions. Described below is the revised revision of the manuscript based on your recommendations detailed below. Corrections and text that were added to the manuscript are shown in red font in the main document and the Supplementary Material.

1. Reviewer’s comment:

**“Reviewer #2: The authors study a mutualistic system by numerical simulations, where the leakers themselves benefit with enhanced growth rate. The rationale behind such behavior was presented in their previous publication (ref. 32) on this subject. Here they study the behavior of such a system, its domain of emergence and stability. Under the assumption of existence of benefit for the leakers, they show that a complex system of leakers and consumers may be stable and diverse.**

**The paper is relevant for a broader scientific community, since it advances our understanding of the mechanisms of symbiotic relations in the context of the Black Queen Hypothesis theory. I would like to recommend the paper for publication, given that the authors address my two comments.**

**1. In the Discussion section they write “Although a well-mixed environment and passive diffusion were considered in our model, the ideas of leak advantage and leaker-consumer mutualism do not depend on the spatial structure and the means of transport. Our theory is thus applicable not only to marine microbial communities (46, 47) and (synthetic) communities in chemostats or test tubes (17, 22, 48) but also to microbial communities in structured environments such as biofilms (49) and soil (5).”**

**I am not convinced that the authors have provided evidence in this work that “the ideas of leak advantage and leaker-consumer mutualism do not depend on the spatial structure and the means of transport.” This may not be true, and I would advise that the authors change this statement in a way that would convey that this is their expectation, for which they have not shown evidence.”**

Response:

Following your comment, we have toned the concerned statement down.

We have clarified that this claim is currently our expectation and understanding based on our present study (see lines 288-290).

2. Reviewer’s comment:

**“2. In the second to last paragraph of the Discussion section starting with “Finally, let us discuss whether a leak advantage would be eliminated through the course of evolution…” they claim that “it would be difficult to reach such an optimized growth state without leakiness, through evolutionary optimization in enzymatic activity.”**

**Again, as in point 1, I am not convinced that this necessarily follows from this work, nor that the authors have shown any evidence for this conclusion. They say that growth optimization that would eliminate leakage is impossible because “Before such isolated optimization is reached, other cells that consume secreted chemicals could either emerge through mutation or invade from elsewhere, there enhancing the growth of the leaker species.”**

**These processes certainly may happen, but not necessarily so, depending on the rates of mutations and/or invasions. It feels to me that this specific question deserves a whole study on its own, and would advise the authors to treat it accordingly.”**

Response:

Following your comment, we have toned the concerned statement down by using hedge words.

We have clearly stated that the described process is just a possibility or hypothesis, and whether it certainly happens or not will be investigated in the future (see lines 374-380).

**To Reviewer #3**

We would like to thank you for your constructive comments and suggestions. Described below is the revised revision of the manuscript based on your recommendations detailed below. Corrections and text that were added to the manuscript are shown in red font in the main document and the Supplementary Material.

1. Reviewer’s comment:

**“In this manuscript, Yamagishi et al. tackle an important problem of species coexistence in microbial ecosystems. They hypothesize that leakage of useful metabolites may be advantageous to the leaker as well as the receiver and using simulations show that leakage can indeed lead to community co-existence.**

**While their simulations are technically sound, I have several major issues with the correspondence of their model with real bacteria and therefore the validity of their conclusions. Prior to publication, the authors should address these concerns. Below, I point out my concerns.**

**1. The authors claim that if the leaked metabolite is not consumed, its concentration in the environment will increase and this will be detrimental to the leaker. However, no intuitive mechanism is presented for this detriment. I looked up their PRL 2020 to find out how this happens. The way I understand it is as follows: The leak advantage stems from the fact that the leaked metabolite (M1 in Fig. 2A) produces its own enzyme E which in turn reroutes the overall flux of the substrate S towards the leaked metabolite and away from biomass production. Therefore, it is advantageous for the organism to maintain low concentrations of M1 in the intracellular medium. Given that the intracellular and the extracellular medium are connected through a diffusion term, it is consequently advantageous to keep a low concentration of M1 in the extracellular medium as well. It appears to me that the symbiosis observed in their model relies on this mechanism therefore it is important to justify that this mechanism is indeed true.**

**Can the authors point to papers where excess of internal metabolites (for example amino acids) leads to production of excess enzymes and therefore a lowered growth rate? If so, they should cite these papers.**

**The authors at least should provide an intuitive explanation about why the leaker is at advantage (the way they did in their PRL paper) ”**

Response:

Thank you for your comments. Please consider our response below.

(i) For leak advantage to function, enzymes do not need to be “in excess.” When some metabolite (a reactant of some multi-body reaction) is involved in an autocatalytic module or branched pathway in the reaction network, leak advantage of such a metabolite can appear. Indeed, even reaction networks with only metabolites can show leak advantages (see newly added Fig. S1).

(i’) Notably, the intracellular metabolism (for cells that can grow) naturally includes (a lot of) autocatalytic modules (see e.g., Blokhuis, *et al.* PNAS 2020) (that can cause excess of some metabolites, and possibly, enzymes). With such autocatalytic modules and branching pathways “flux control mechanism” can generally work.

(ii) Even though the detailed mechanisms might be unclear, the accumulation of metabolites, that are normally useful at low concentrations such as organic acids in a medium, hinders cell growth (see example, ref. 9 in the manuscript, which reported that the growth of organic acid leaking cell species can be promoted by the existence of another species that consumes the accumulated organic acids).

(iii) We mainly mentioned the more intuitive flux control mechanism (out of the two mechanisms proposed in our PRL 2020) because the underlying mechanisms of leak advantage is independent of whether leaker-consumer mutualism can appear or not. In contrast, we also proposed “growth-dilution mechanism.” This mechanism is generally caused by the balance between chemical synthesis and growth-induced dilution, and thus, is expected to be more likely and inevitable to appear.

Following your suggestion, we have added a remark reflecting the above points and an explanation about leak advantage to the Introduction and the Results in the revised manuscript (as in our PRL 2020) (lines 64-65 and 131-132).

2. Reviewer’s comment:

**“2. The authors assume that some of the reactions in the model are reversible (diffusion across the membrane) while others are irreversible S -> M1.**

**All biological reactions are essentially reversible. Importantly, the increase in S -> M1 flux that is presumably responsible for lower growth rate under conditions of high M1 may not be realized in a thermodynamically balanced situation where a high M1 may automatically decrease the S to M1 flux.**

**As a broad comment, the authors should address the lack of thermodynamic equilibrium in their model and how introducing thermodynamic equilibrium may affect their phase diagrams. ”**

Response:

In the present paper, we consider cells with growth (and division) which themselves are non-equilibrium systems. Thus, the system in concern cannot be at equilibrium. [If we cut-off all in/outflow of chemicals (including nutrients) and make every reaction reversible, the system would finally reach thermodynamic equilibrium (probably after a long time; see e.g., Awazu and Kaneko PRE 2009), but it is out of the scope of the present study, since we are interested in living cells (with growth).]

In contrast, we can introduce the reversibility of chemical reactions to our models. We then confirmed that the reversibility of chemical reactions does not qualitatively alter our theory. In newly added Fig. S2, we showed that a simple network (that shown in Fig. 2) with reversible chemical reactions (S+E→M_1_+E and S→M_2_) can also exhibit the leak advantage and leaker-consumer mutualism. (Note that, even in this setting, the concerned system is not at thermodynamic equilibrium because the net flux from S to BM exists and thereby the cell is steadily growing.)

We have referred to this new result in the Results (see lines 132-134 and newly added Fig. S2).

3. Reviewer’s comment:

**“Minor concerns:**

**1. The authors should provide a notation table. There are many quantities and it would be easy to follow the paper if the notations were clarified at the start ”**

Response:

Following your suggestion, we have added a notation table (see Table 1).

4. Reviewer’s comment:

**“2. Fig. 3 caption mentions rho which is only defined in the appendix”**

Response:

Following your comment, we have added an explanation on the parameter ρ.

5. Reviewer’s comment:

**“3. Typo on line 129 ”**

Response:

Thank you for your careful reading. We have fixed the typos (from “S\to E” to “S\to M_1”).
